# Main substance of consumption of patients in follow-up in the hospital consultation. Have there been changes in the current pandemic context? And what about women?

**DOI:** 10.1192/j.eurpsy.2022.2219

**Published:** 2022-09-01

**Authors:** A. Pérez Oms, F. Dinamarca, F. Casanovas, C. Llimona Sánchez, D. García Hernández, M. Calls Samora, J.M. Ginés, J. Mayans, V. Pérez-Solà, F. Fonseca, M. Torrens

**Affiliations:** 1 Parc de Salut Mar, Instituto De Neuropsiquiatría Y Adicciones (inad), Barcelona, Spain; 2 Parc de Salut Mar, Institut De Neuropsiquiatria I Addiccions (inad), Barcelona, Spain; 3 Parc de Salut Mar, Institut De Neuropsiquiatria I Addiccions, Barcelona, Spain; 4 Autonomous University of Barcelona, Psychiatry, Barcelona, Spain

**Keywords:** Covid-19, sex differences, women

## Abstract

**Introduction:**

*Gender is a factor influencing characteristics of substance use disorders. The Covid-19 pandemic has had a great impact in all areas of society, meaning a context of exceptionality in this population. Usually the male population represents a greater number of patients in general samples, so the descriptive characteristics of a global sample may not be representative in the case of women.*

**Objectives:**

*- Identify if there are changes in the main substance of psychoactive substance use during the Covid-19 pandemic. - Identify possible divergences in characteristics of the general sample with respect to the sample made up of women.*

**Methods:**

It will be used data collected in the database of patients in follow-up with the addiction consultation service in two periods of 6 months, one prior to the pandemic situation due to Covid-19 and another corresponding to same period in 2020. A descriptive analysis is carried out by applying chi-square statistic, performing the analysis by subgroups according to gender.

**Results:**

84.8% of total sample are men. Results show that there are no statistically significant differences between periods in main substance of consumption. Despite this, differential trends can be observed in the sample that correspond to women with respect to the global sample and that of men.

**Conclusions:**

Taking into account the low number of women that make up the sample, the fact that differential trends are observed could indicate possible differences, which in case of increasing the sample size could acquire statistical significance and that this it would be specific to women subgroup.

**Disclosure:**

No significant relationships.

